# Effects of Obesity and Fall Risk on Gait and Posture of Community-Dwelling Older Adults

**Published:** 2019

**Authors:** Thurmon E. Lockhart, Christopher W. Frames, Rahul Soangra, Abraham Lieberman

**Affiliations:** 1 School of Biological and Health Systems Engineering, Arizona State University, Tempe, AZ,85281, USA; 2 Barrow Neurological Institute, Phoenix, AZ, USA; 3 Crean College of Health and Behavioral Sciences, Chapman University, Irvine, CA,92618, USA; 4 Fowler School of Engineering, Chapman University, Orange, CA, 92866, USA

## Abstract

Epidemiological studies link increased fall risk to obesity in older adults, but the mechanism through which obesity increases falls and fall risks is unknown. This study investigates if obesity (Body Mass Index: BMI>30 kg/m^2^) influenced gait and standing postural characteristics of community dwelling older adults leading to increased risk of falls. One hundred healthy older adults (age 74.0±7.6 years, range of 56–90 years) living independently in a community participated in this study. Participants’ history of falls over the previous two years was recorded, with emphasis on frequency and characteristics of falls. Participants with at least two falls in the prior year were classified as fallers. Each individual was assessed for postural stability during quiet stance and gait stability during 10 meters walking. Fall risk parameters of postural sway (COP area, velocity, path-length) were measured utilizing a standard forceplate coupled with an accelerometer affixed at the sternum. Additionally, parameters of gait stability (walking velocity, double support time, and double support time variability) were assessed utilizing an accelerometer affixed at the participant’s sternum. Gait and postural stability analyses indicate that obese older adults who fell have significantly altered gait pattern (longer double support time and greater variability) exhibiting a loss of automaticity in walking and, postural instability as compared to their counterparts (i.e., higher sway area and path length, and higher sway velocity) further increasing the risk of a fall given a perturbation. Body weight/BMI is a risk factor for falls in older adults as measured by gait and postural stability parameters.

## 1. INTRODUCTION

Obesity is rising at an alarming rate for older adults (Flegal, 2010; Mokdad, 2001; Cynthia L. Ogden et al., 2006; Y. Wang & Beydoun, 2007). The prevalence of obesity (BMI>30 kg/m^2^) is higher in older adults aged 60 and over approximately 37% more than that in younger adults (C. L. Ogden, Carroll, Fryar, & Flegal, 2015). Obesity is also found to be associated with risk factors for various health conditions (e.g., cardiovascular diseases, stroke, and diabetes) (Bray, 2004; Kopelman, 2000; Strazzullo et al., 2010; T. J. Wang, 2004; Wilson, D'Agostino, Sullivan, Parise, & Kannel, 2002). Obese individuals are found to have higher chances of sustaining injuries compared to their lean counterparts (Finkelstein, Chen, Prabhu, Trogdon, & Corso, 2016). Falls have been identified as one of the most common causes of injury (36% of all injuries) in the obese (Matter, Sinclair, Hostetler, & Xiang, 2007), and obese older adults fell almost twice as frequently (27%) as their lean counterparts (15%) (Fjeldstad, Fjeldstad, Acree, Nickel, & Gardner, 2008).

Although epidemiological studies link increased fall risk to obesity in older adults, the mechanism through which obesity increases falls and associated fall risks is lacking. Indeed, an array of obesity-related physiological and biomechanical factors induce postural/gait alterations that may increase fall risks. For example, obesity is associated with an anterior displacing of the whole-body Center of Mass (COM) and thus influencing trunk posture while standing and walking. This impairs both static and dynamic stability(Kejonen, Kauranen, & Vanharanta, 2003; Spyropoulos, Pisciotta, Pavlou, Cairns, & Simon, 1991). Obesity is also found to be associated with a wide range of musculoskeletal conditions (Anandacoomarasamy, Caterson, Sambrook, Fransen, & March, 2007) that may influence bodily movement and postural stability (Compston et al., 2011; Flegal, Williamson, Pamuk, & Rosenberg, 2004; Handrigan, Corbeil, Simoneau, & Teasdale, 2010; Hills, Hennig, Byrne, & Steele, 2002) leading to more falls. Furthermore, additional postural and gait control constraints (on gait and posture) associated with obesity are largely unidentified (Corbeil, Simoneau, Rancourt, Tremblay, & Teasdale, 2001; Hue et al., 2007).

In this study, to quantify the static and dynamical properties of stability and provide further insights into postural/gait control and obesity, several gait and postural measures were adopted. Wearable technology provides a means to improve the accessibility of routine analysis of gait and posture in assessing fall risk of an individual, thus employed a wearable wireless accelerometer-based gait and postural analyses system (along with the traditional forceplate). We hypothesized that obesity will influence postural and gait stability and increase falls and fall risks.

## 2. METHODS

One hundred older community-dwelling volunteers (56 to 90 years, mean age 74.3±7.6 years) participated in this study. This sample size was selected to provide a reliable confidence interval in classifying fallers and non-fallers (Bartlett, Maki, Fernie, Holliday, & Gryfe, 1986; Błaszczyk, Cieślinska-Świder, Plewa, Zahorska-Markiewicz, & Markiewicz, 2009; Teasdale et al., 2006). Study participants are divided into two groups based on their BMI: non-obese (BMI<30 kg/m^2^) and overweight/obese (BMI>30 kg/m^2^) (Bartlett et al., 1986). Participants’ history of falls were recorded for the previous 2 years, with emphasis on frequency and characteristics of falls. Any person with at least two falls in the prior year was classified as a faller and the others as non-faller. Falls were characterized as all four limbs or the buttocks striking the ground without loss of consciousness. Individuals who were demented, legally blind, had a history of stroke with hemiparesis or multiple strokes, Parkinson disease, orthostatic hypotension, alcoholism, drug dependency (including benzodiazepines) were excluded (2 out of 100 excluded). Participants were recruited through advertisements at community centers. No monetary compensation was provided. Participants' anthropometric data is presented in Table 1.

**Figure 1.**
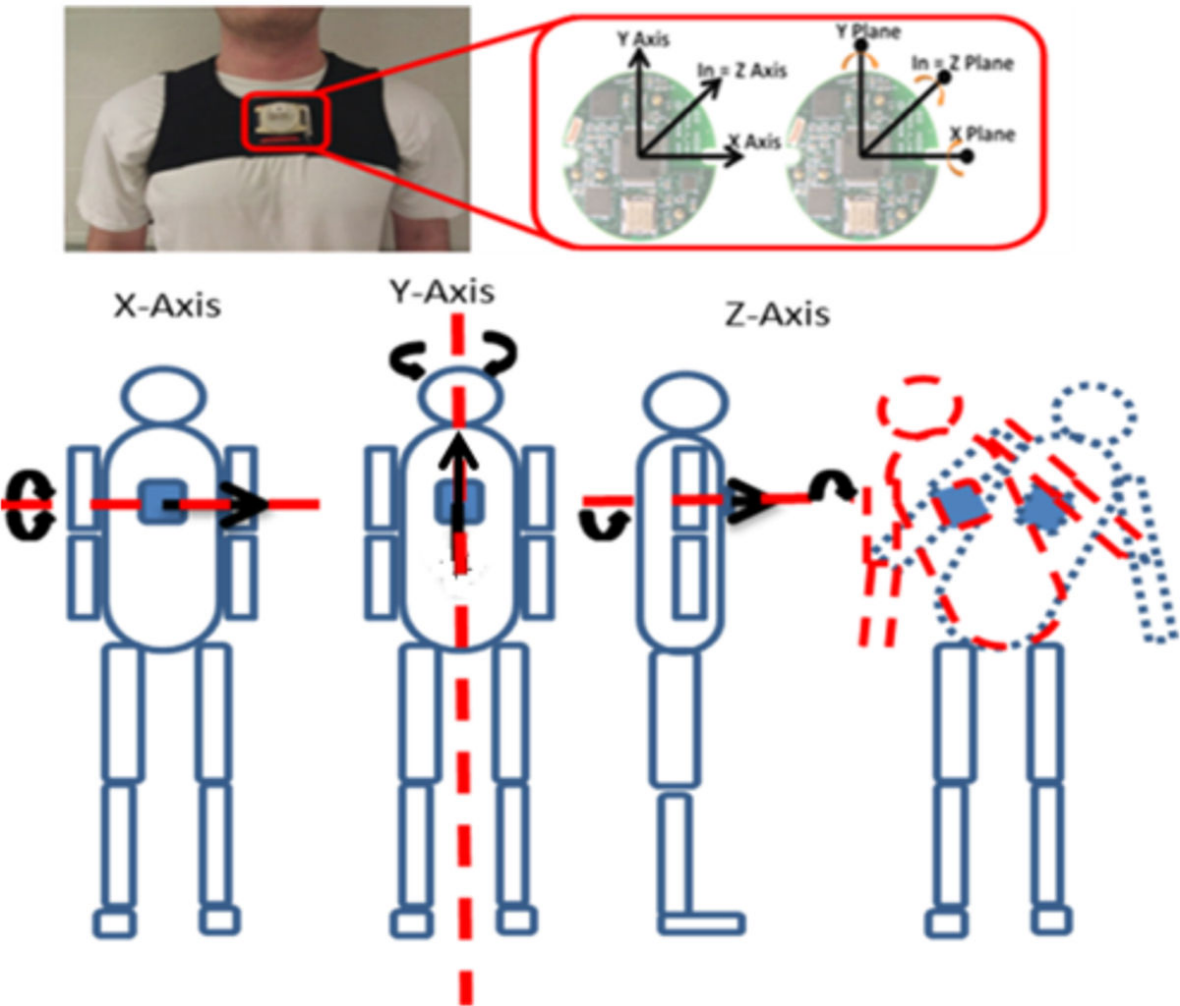
IMU placement and axis of motion capture.

The study took place in four different community centers (Dale City, Woodbridge, Leesburg, and Manassas) located in northern Virginia, using the same set of instruments (i.e. inertial measurement unit (IMU) and a forceplate) on four different days. The IMU system consists of MMA7261QT tri-axial accelerometers and IDG-300 (x and y plane) and ADXRS300A (z plane) uniaxial gyroscopes (Figure 1).

These sensors collected linear acceleration and angular rate at 128 Hz, providing six degrees of freedom motion capture (Lockhart, Soangra, Zhang, & Wu, 2013). The data acquisition was carried out using a Bluetooth adapter and laptop via a customized LabView program. A stopwatch was also used to time the 10-meter walking completion time. Forceplate was used for assessing postural stability at a sampling rate of 1200 Hz (BETEC #K80102, Type 45550–08, Bertec Corporation, OH 43212, USA).

This study was approved by the Virginia Tech Institutional Review Board (VT-IRB) and was conducted in collaboration with the Northern Virginia Fall Prevention Coalition (NVFPC) and INOVA Hospital. All older adults provided written consent for participation. They wore comfortable attire and their foot placement was standardized while standing on the forceplate while wearing an IMU affixed to their sternum. All measurements were performed barefoot in quiet standing, looking in the forward direction, and with their arms by their sides. Participants were asked to stand with eyes open. Each measurement lasted 60 seconds and was repeated twice in all participants. Gait characteristics were observed using a clinically validated (Scivoletto et al., 2011) gait assessment tool during a 10-meter walk utilizing an accelerometer affixed to the sternum. Rest of 3 minutes was provided between each measurement. BMI was calculated for each participant based on his/her height and weight. The Activities-specific Balance Confidence (ABC) scores were collected during the testing session. Participants indicated their level of confidence in doing the activity without losing balance or becoming unsteady from choosing one of the percentage points on the scale from 0% to 100%. If the participants did not perform the activity in question, they tried to imagine how confident they could be if they had to do the activity.

Gait event times were identified using an inertial measurement unit (IMU) positioned over the sternum (McCamley, Donati, Grimpampi, & Mazzà, 2012). A modified continuous wavelet transform (CWT) method previously reported by McCamley et al., 2012, was utilized as a gait detection algorithm (McCamley et al., 2012). The wavelet transforms support time-frequency decomposition of non-stationary signals and does not require preprocessing of the signal, making it ideally suited for a peak detection algorithm (Moe-Nilssen, 1998).

**Figure 2.**
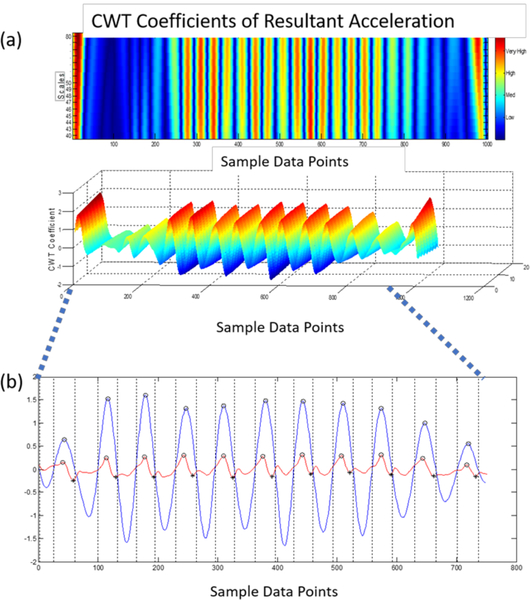
a) Detection of HC events using the CWT differentiation method. b) Peaks (blue) equate to HC events; the local minima in the AP acceleration (red) equate to TO events.

The resultant acceleration, a signal invariant to axis alignment, was analyzed to mitigate any alignment errors reliant on IMU placement. Furthermore, due to the placement of the inertial sensor, the Gaussian (gaus1) mother wavelet used in McCamley et al., 2012, was deemed inappropriate for the inertial data. Instead, a symlet (sym4) mother wavelet with an order of 4 and a scale between 35–70, was employed over the resultant acceleration signal to detect the instant events. Heel contacts (HC) were identified as the maxima of the CWT differentiated signal. The toe off (TO) events, however, were processed by a windowing technique in which the HC points and the subsequent zero crossings of the CWT differentiated signal determined an appropriate window size where the instant of the first minimum in the AP acceleration signal was considered a TO event (González, López, Rodriguez-Uría, Álvarez, & Alvarez, 2010; Zijlstra & Hof, 2003). Given the placement of the inertial sensor and the extracted resultant acceleration, the CWT method previously employed, in which the maxima of a further CWT differentiated signal was considered the final contact event, could not be relied upon to determine the TO time. Moreover, because of the inherent gait deficiencies associated with the community-dwelling population of older adults and the intermittent “shuffling of gait,” a window detection method similar to Gonzalez et al., 2010, was better suited for the extracted signal (González et al., 2010). Finally, the right and left HC events were designated by the sign of the vertical angular velocity at the instant of the first HC in which every other HC equated to a stride (McCamley et al., 2012). The signal was preprocessed with a 4^th^ order low pass Butterworth filter and a cutoff frequency of 2 Hz (Zijlstra & Hof, 2003) (Figure 2).

A two-way (weight status × fall risk) between-subject ANOVA was performed with “weight status” (obese and non-obese) and “fall risk” (fallers and non-fallers) as the independent variables on gait and posture parameters as the dependent variables using JMP (JMP®, Version 13. SAS Institute Inc., Cary, NC, 1989–2007). Table 2 and Table 3 show the computed gait and postural stability parameters.

## 3. RESULTS

Significant differences were observed in a multitude of postural and gait stability measures comparing obese/non-obese fallers and non-fallers (Table 4).

***ABC scores*** were significantly different between fallers (51.8%) and non-fallers (81.5%) (F_1,36_ = 23.92; p< .0001), however, no significant differences were observed for obese and non-obese fallers and non-fallers. Similar scores were also observed for older adults at risk of falling (Lajoie & Gallagher, 2004) - with an ABC score of less than 67% being predictive of future falls. ***Fall frequency*** was significantly different among obese and non-obese fallers and non-fallers. In this test population, fallers fell significantly more (average 3.23 falls) (F_1,94_ = 527.24; p< .0001) than their non-falling counterpart (0.14 falls) and obese individuals fell significantly more (2.2 falls) (F_1,94_ = 58.84; p< .0001) than non-obese counterpart (1.17 falls).

**Figure 3.**
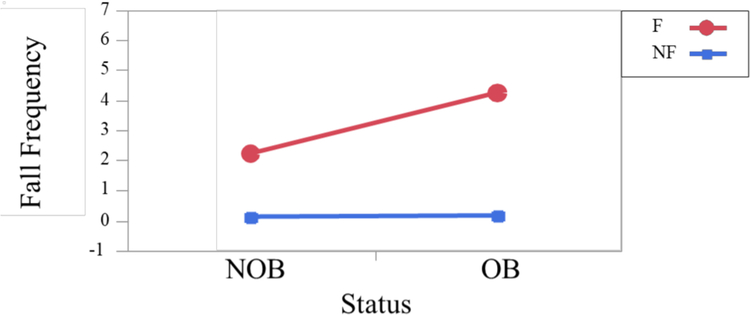
Obese fallers experience more falls than non-obese fallers.

Furthermore, obese fallers fell significantly more (4.25 falls) (F_1,94_ = 54.30; p< .0001) than their non-obese fallers (2.2 falls) (Figure 3).

Significant differences were observed in a multitude of postural stability measures between fallers and nonfallers as well as obese and nonobese individuals: Sway area (ellipse area, p=0.0012, F_1, 93_=11.18; circular area, p<0.0048, F_1, 93_=8.34), mean velocity (p=0.016, F_1, 93_ = 6.00), and mean path length of COP (p=0.01, F_1,93_ = 6.13).

**Figure 4.**
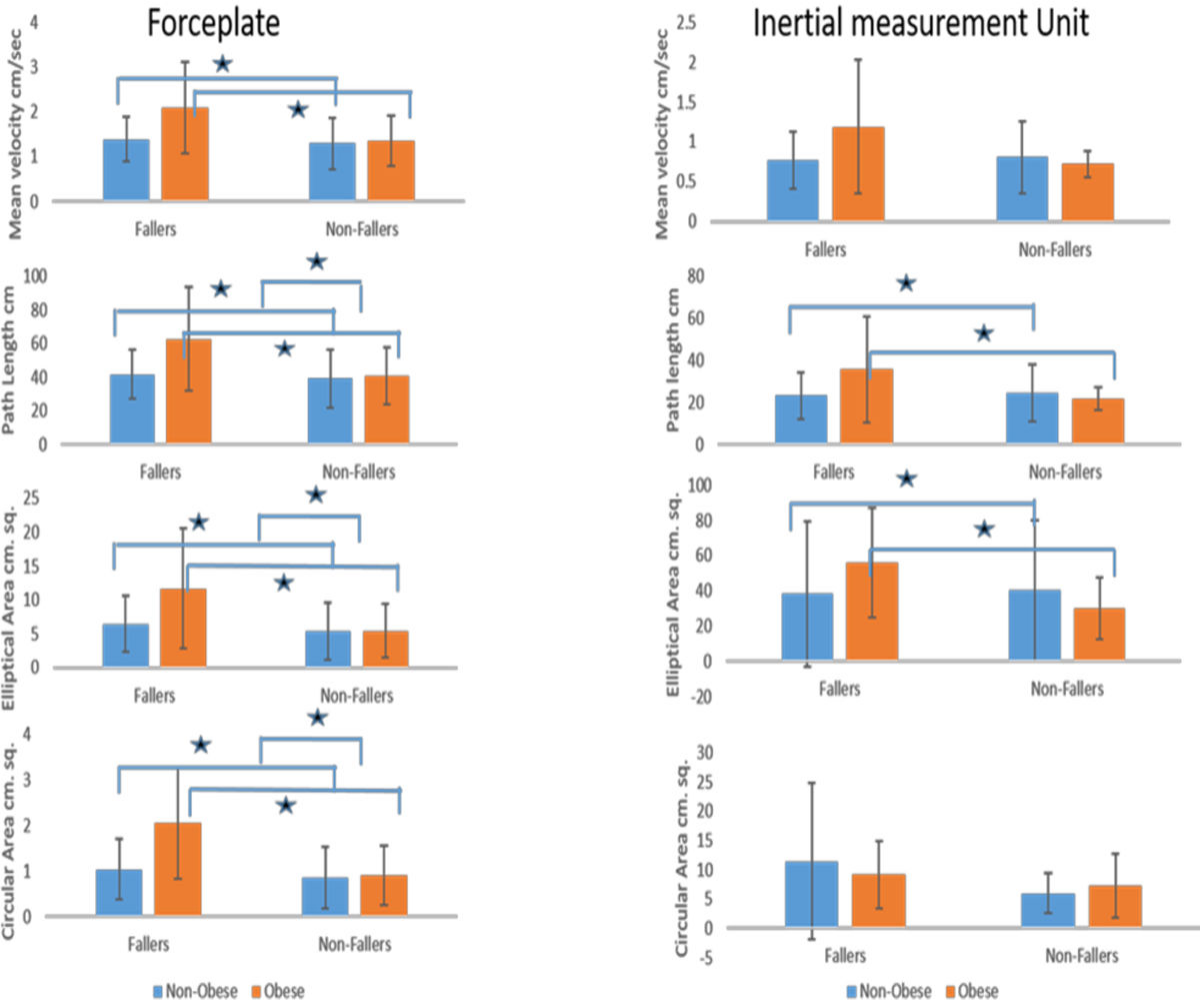
Comparative graph showing center of pressure (COP) circular area, COP elliptical area, COP path length and COP velocity from both instruments a) forceplates and b) IMU.

Similar results were obtained for IMU postural stability measures the ellipse area. Sway area (ellipse area, p=0.015, F_1, 93_=6.13; mean velocity (p=0.016, F_1, 93_=6.00), and mean path length of COP (p=0.04, F_1,93_=4.32) (Figure 4).

**Figure 5.**
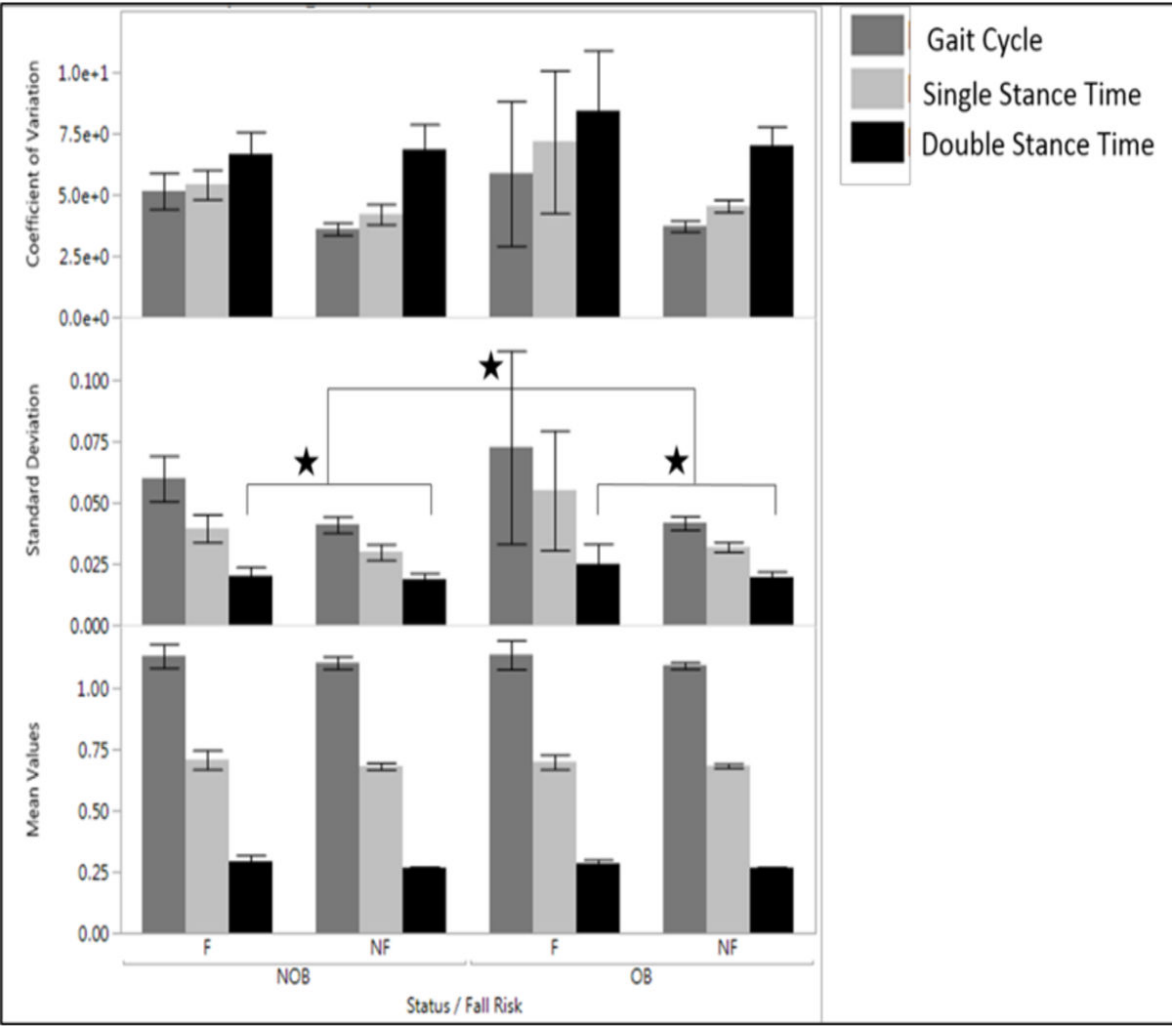
Gait Cycle Time, double stance time and single stance time of Obese Fallers/Non-Fallers and Non-Obese participants and their linear variabilities such as standard deviation and Coefficient of variation **Note:** Melzer et. al. using forceplates reported the elliptical area in fallers as 6.7±0.7 cm sq. and 5.6±0.3 cm sq. for nonfallers (Melzer, Benjuya, & Kaplanski, 2004). COP velocity was found as 2.4±0.1 cm/sec among fallers and 1.9 ±0.1 cm/sec among non-fallers(Melzer et al., 2004). The COP path length was found to be 47.3±2.8 cm in fallers and 38.9±1.1 cm in non-fallers. For the IMU: Similar values were reported using accelerometers, the sway velocity was found to be 3.5 cm/sec for eyes open condition in healthy individuals(Mayagoitia, Lotters, Veltink, & Hermens, 2002) and 1.5±0.9 cm/sec in fallers and 1.2±0.7 in nonfallers(Doheny et al., 2012). The sway path length was reported as 25.5±16.7 cm in non-fallers and 31±20.5 cm in fallers(Doheny et al., 2012).

In general, regarding the gait variables, double support time was significantly different between fallers and nonfallers (p=.0008, F_1, 87_ = 11.98), and obese and nonobese (p=.001, F_1, 87_ = 5.70), obese fallers having a significantly longer duration of DST indicating gait adaptation. Variability of DST was also significantly different between fallers and nonfallers as well as obese and nonobese with the greatest variability for obese fallers (p=.05, F_1, 88_ = 3.75) (Figure 5). Walking velocity was significantly different for fallers and nonfallers (p=.01, F_1, 87_ = 6.44).

## 4. DISCUSSION

The present study investigated the effects of obesity and fall risk on gait and posture of community-dwelling older adults using biomechanical analyses on signals acquired from forceplate and IMU systems. The results indicated that body weight/BMI is a risk factor for falls in older adults as measured by gait and postural stability parameters and that accelerometer-based postural and gait stability analyses could be used as objective measures of fall risk and postural and gait instability.

The assessment of gait patterns (e.g., walking velocity and double support time) during walking provides pertinent information regarding dynamic stability of walking and provides an effective tool for evaluating and quantifying gait problems associated with fall-prone individuals.

Previous studies indicated that an individual’s inability to walk in a repetitive and stable manner is regarded as a possible sign of an evolving gait disorder leading to falls (Hausdorff, Rios, & Edelberg, 2001). For example, a study investigating the gait characteristics of older adults who were hospitalized after falls (Guimaraes & Isaacs, 2009) suggested that individuals with step variability fell more often than non-fallers. The work of Imms and Edholm (Imms & Edholm, 1979) also demonstrated that gait variability is linked to falls in late life. Although many older adults walk without any noticeable gait impairment (Bloem et al., 2016), Isaacs proposed that one of the effects of aging is an increased intercycle (step-to-step) variability of gait, possibly associated with the gradual deterioration of balance mechanisms that are known to occur (Der Wiel et al., 2002). Two facets (of gait characteristics) related to postural stability are walking velocity and double-support time (temporal). The decrease in walking velocity and increase in double-support time will lead to greater stability and may be regarded as compensation for instability. However, an increase in the variability of double support time may indicate a lack of compensation for instability (a loss of automaticity in walking) and predispose an individual to falls, especially when balance mechanisms are stressed (Hausdorff et al., 1997). The metabolic costs associated with stance phase dynamics indicate that much of the metabolic cost of human walking is attributed to transitions occurring during the stance phase of the gait cycle (Umberger, 2010). Indicating that obese adults who fall in this study adopted a conservative strategy to maintain stability while increasing the double support time at a greater metabolic cost.

A significant increase in sway parameters (circular area, ellipse area, and path length) were also observed in obese fallers. Traditionally, greater COP displacements have been linked with less stability and, consequently, increased fall risk. This implies the motor system was unable to adjust to the demands inherent in obesity during stance, resulting in diminished adaptability and stability. In this context, the increase in sway area and path length may be a result of impaired feedback control or impaired proprioception/vision/vestibular system leading to a reduced adaptive capacity of the postural system (Manor et al., 2010). Moreover, the firing of postural muscles may follow an adaptive strategy to reduce joint loads in obese older individuals that diminish postural stability. From a biomechanics perspective, it may also be due to the inability of older people to control and accelerate the whole-body center-of-mass (COM) over the base of support, perhaps due to lack of strength and degradation of type II fibers in skeletal muscles (Delmonico et al., 2009). While muscle strength was not objectively measured in this study, it has been documented that many older people have relatively weaker tibialis anterior and vastus lateralis muscle strength compared to that of healthy adults (Hurley, Rees, & Newham, 1998; Murray, Gardner, Mollinger, & Sepic, 1980) making them more susceptible to falls. Obesity is often related to a lower level of physical activity, impaired cardiorespiratory fitness and knee strength compared to non-obese individuals (Duvigneaud et al., 2008), possibly impairing the ability to correct for a shift in the body’s center of mass and prevent falling. Increased postural sway could be an adaptive strategy to provide additional stability under conditions of weakness in muscles involved in postural control. Age-related deterioration of sensory and neuromuscular control mechanisms could add to this problem. Degradation of balance shows that fall risk is increased in persons with higher BMI.

Obese older adult fallers and non-fallers have a larger number of risk factors for falling. Yet a smaller percent (8/56–14.2%) of obese persons versus non-obese persons (9/25 – 36%) fell in this study. The number of falls experienced prior to the study was retrospectively estimated. Such estimates based on memory are not as accurate as obtained through a prospective study of actual falls – which we believe will reveal a higher percent of actual falls in obese individuals. Moreover, we do not know the BMI state of the participants when they fell in the past. A prospective study of actual fall and the BMI state will clarify this role.

In order to provide a low-cost, objective assessment of fall risk, accelerometry using microelectromechanical systems (MEMS) technology has been employed to assess the gait and postural stability parameters. These measures facilitate long-term monitoring of activity of daily living using wearable sensors (Bouten, Koekkoek, Verduin, Kodde, & Janssen, 1997; Mathie, Celler, Lovell, & Coster, 2004; Mathie, Coster, Lovell, & Celler, 2003). Accelerometers are desirable in monitoring human postures since accelerometers respond to both frequency and the intensity of movements. This has enabled the development of a small, lightweight, portable system that can be worn by a free-living subject without motion impediment. Using this system, researchers can acquire indirect measures of metabolic energy expenditure (Handrigan et al., 2010; Kejonen et al., 2003), detect falls and reliably discriminate body posture (Bouten et al., 1997; H. B. J. Bussmann, Reuvekamp, Veltink, Martens, & Stam, 1998) with varied accuracy with recognition rates of 85% to 95% for ambulation and posture (J. B. J. Bussmann et al., 2001; Fino, Frames, & Lockhart, 2015; P. C. Fino et al., 2015; Lockhart et al., 2013).

## 5. LIMITATIONS

The older participants were aware that they were participating in a fall risk assessment protocol. This could be a bias in the population studied. They may be conscious of the environment and their performance may have been affected by the environment. We tested the balance of community-dwelling older adults in four different community centers, and the environment of data collection may also have been a confound in this study.

The age range of participants in this study is fairly large 56–90 years. Aging influences fall-risk and we previously reported that aging influences biomechanics of slips and falls (Lockhart, Woldstad, & Smith, 2003). We have also reported earlier that sensitive motor control measures like dynamic stability can differentiate fall-prone older individuals from age-matched healthy older adults(Lockhart & Liu, 2008). Aging in human beings affects neuromuscular intactness, sensory degradation, muscle atrophy, vision and vestibular loss which play important role in assessment of fall risk. Although there is great heterogeneity in the health outcomes of older individuals (Lowsky, Olshansky, Bhattacharya, & Goldman, 2014). Some individuals appear frail and require assistance in daily routines already in their 60’s and 70’s whereas others remain independent of assistance until very extreme ages. Thus, biological age as a major predictor of fall risk rather than chronological age(Jylhava, Pedersen, & Hagg, 2017).

The community center setting in which data were obtained for this study provided a familiar environment for the older participants. At the same time, the non-laboratory setting limited the scope of this data. Howsoever, such analyses may provide insight as to the potential fall risk associated with older obese participants.

## 6. CONCLUSION

Obesity in older adults is undoubtedly recognized as an important issue with fall risk implications. However, little is known about the relationship between obese older persons and their gait characteristics (especially the fallers). The key finding of this study are: 1) postural and gait stability is compromised in obese fallers; 2) acceleration-based gait and postural parameters are able to distinguish between obese fallers and non-fallers.

The present study suggests that the body-weight influences postural balance and gait stability in obese older individuals utilizing traditional biomechanical parameters.

Inertial sensors can be helpful to detect fall risk caused by higher body mass in older individuals. Indeed, our findings indicate that a change in temporal variability can detect postural changes due to obesity in older persons and IMUs may serve as an alternative instrument in assessing this vital information relevant to one’s dynamic stability and fall risks.

## Figures and Tables

**Table 1.** Anthropometric data for obese/non-obese faller/non-faller participants.

	Non-Obese		Obese	
	Faller	Non-Faller	Faller	Non-Faller
Age (years)	76.82±6.87	77.41±8.49	72.29±4.72	72.68±7.40
Height (m)	1.71 ±0.06	1.67±0.11	1.61 ±0.07	1.64±0.05
Weight (kg)	79.14±8.18	67.26±12.41	80.77±21.98	87.66±21.05
BMI	26.85±2.08	24.29±2.16	31.27±8.09	32.65±7.62

**Table 2.** Definition of gait stability parameters using IMU.

Gait Parameter	Definition
**Gait Cycle Time (s)**	Time elapsed between two consecutive heel contacts of the ipsilateral foot.
**Step Time (s)**	Time elapsed from the heel contact of one foot to heel contact of the subsequent contralateral foot.
**Single Support Time (s)**	Time elapsed from the heel contact to the toe off of a single footfall.
**Double Support Time (s)**	Time elapsed from the heel contact of one foot to the toe off of the contralateral foot. It is the sum of two periods of double support in the gait cycle.
**Swing Time (s)**	Time elapsed between toe-off of a gait cycle to the subsequent heel contact of the same foot.
**Gait Speed (cm/s)**	Total distance walked divided by duration of walk.
**Root Mean Square (RMS *norm*)**	Statistical measure of the trunk acceleration magnitude in the AP, ML, or V direction compared to the total trunk acceleration magnitude.
**Coefficient of Variation (CV)**	Measure of variability normalized to the mean of a specific gait parameter. CV = (SD/Mean) × 100

**Table 3.** Definition of postural stability parameters using forceplate and IMU.

Postural Stability Parameters	Definition
**COP Path Length**	Distance covered by COP over a certain time period by summing the Euclidean distance between the points: $Path\;Length=\sum_{n-1}^{N}\sqrt{{\left(x_{n}-x_{n-1}\right)}^{2}+{\left(y_{n}-y_{n-1}\right)}^{2}}$ Where x, y are the coordinates of COP and N is the number of data points
**Elliptical Area**	Elliptical Area is computed as a 95% confidence ellipse around the area covered by COP, computed using the eigenvalues of the variance/covariance matrix
**COP Velocity**	COP velocity is calculated through the displacement of the total sway of the COP in both directions (AP and ML) divided by the total duration of the trial
**COP Circular Area**	After detrending the COP-AP and COP-ML signals, mean sway radius is obtained $r=\left(\sqrt{x^{2}+y^{2}}\right)/n$ *Circular Area* = *πr*^2^

**Table 4.** Results of ABC score, Fall Frequency and, gait and postural stability measures. Postural stability measures are provided for both forceplate/IMU derived measures during quite stance with eyes open condition. Gait parameters were derived using an IMU.

Fall Risk	Fallers		Non-Fallers	
Weight Status	Non-Obese	Obese	Non-Obese	Obese
ABC	49.98±26.26	51.93±24.88	81.86±16.15	81.20±11.98
Fall Frequency	2.22±0.16	4.25±0.17	0.12±0.09	0.16±0.06
**Postural Stability Measures : Forceplate**				
Circulr Area_FP_[cm.sq]	1.04±0.66	2.05±1.22	0.85±0.67	0.91±0.65
Elliptical Area_FP_[cm.sq]	6.43±4.13	11.67±8.87	5.36±4.19	5.42±3.92
Path Length_FP [cm]	41.81±14.83	62.80±30.60	39.23±17.28	40.52±16.93
Mean Velocity_FP_[cm/sec]	1.39±0.49	2.09±1.02	1.30±0.57	1.35±0.56
**Postural Stability Measures : IMU**				
Circular Area_IMU_[cm.sq]	11.47±13.38	9.18±5.79	6.00±3.43	7.28±5.50
Elliptical area_IMU_[cm.sq]	38.22±41.03	56.22±31.13	40.16±39.91	30.08±17.72
Path Length_IM U [cm]	23.28±11.02	35.84±25.25	24.49±13.70	21.89±5.23
Mean Velocity_I MU [cm/sec]	0.77±0.36	1.19±0.84	0.81±0.45	0.72±0.17
**Gait Stability Measures : Inertial Measurement Unit**				
Double Support Time [sec]	0.275±0.073	0.325±0.067	0.266±0.023	0.264±0.025
SD Double support time [sec]	0.021±0.005	0.033±0.349	0.018±0.003	0.018±0.002
Walking Velocity [m/sec]	0.988±0.376	0.926±0.349	1.132±0.277	1.261±0.344

## NOMENCLATURE

BMI: Body Mass Index
COP: *Center of Pressure*
COM: Center of Mass
CWT: Continuous Wavelet Transform
HC: Heel Contact
TO: Toe Off
IMU: Inertial Measurement Unit
DST: Double Support Time
ABC: Activities-specific Balance Confidence
